# A modified fluorescent sensor for reporting glucose concentration in the airway lumen

**DOI:** 10.1371/journal.pone.0254248

**Published:** 2021-07-09

**Authors:** Jade Bearham, Nina Krutrök, Botilda Lindberg, Maximillian Woodall, Annika Astrand, John D. Taylor, Matthew Biggart, Stanislavs Vasiljevs, Robert Tarran, Deborah L. Baines

**Affiliations:** 1 Institute for Infection and Immunity, St George’s University of London, London, United Kingdom; 2 Research and Early Development, Respiratory & Immunology, BioPharmaceuticals R&D, AstraZeneca, Gothenburg, Sweden; 3 Department of Cell Biology & Physiology, University of North Carolina, Chapel Hill, North Carolina, United States of America; Emory University School of Medicine, UNITED STATES

## Abstract

We have modified the periplasmic *Escherichia coli* glucose/galactose binding protein (GBP) and labelled with environmentally sensitive fluorophores to further explore its potential as a sensor for the evaluation of glucose concentration in airway surface liquid (ASL). We identified E149C/A213R GBP labelled with N,N’-Dimethyl-N-(iodoacetyl)-N’-(7-nitrobenz-2-oxa-1,3-diazol-4-yl)ethylenediamine (IANBD, emission wavelength maximum 536nm) with a K_d_ for D-glucose of 1.02mM and a fluorescence dynamic range of 5.8. This sensor was specific for D-glucose and exhibited fluorescence stability in experiments for several hours. The use of E149C/A213R GBP-IANBD in the ASL of airway cells grown at air-liquid-interface (ALI) detected an increase in glucose concentration 10 minutes after raising basolateral glucose from 5 to 15mM. This sensor also reported a greater change in ASL glucose concentration in response to increased basolateral glucose in H441 airway cells compared to human bronchial epithelial cells (HBEC) and there was less variability with HBEC data than that of H441 indicating that HBEC more effectively regulate glucose movement into the ASL. The sensor detected glucose in bronchoalveolar lavage fluid (BALf) from diabetic db/db mice but not normoglycaemic wildtype mice, indicating limited sensitivity of the sensor at glucose concentrations <50μM. Using nasal inhalation of the sensor and spectral unmixing to generate images, E149C/A213R GBP-IANBD fluorescence was detected in luminal regions of cryosections of the murine distal lung that was greater in db/db than wildtype mice. In conclusion, this sensor provides a useful tool for further development to measure luminal glucose concentration in models of lung/airway to explore how this may change in disease.

## Introduction

The development of molecular sensors that respond to real-time changes in the concentration of intra- and extracellular molecules has rapidly increased. Sensors that measure Ca^2+^ [1], lactate [2], glutamate [3] with high affinity and speed have been described and used to image changes in such parameters in several cell types.

We previously employed the periplasmic *Escherichia coli* glucose/galactose binding protein (GBP) labelled with environmentally sensitive fluorophores to investigate its potential as a glucose sensor for the evaluation of glucose concentration in airway surface liquid (ASL). This liquid lines the luminal surface of the airways. In the distal lung the depth of fluid is <1μm, increasing to approximately 7μm in the upper airways and further in nasal secretions. Thus, to estimate glucose concentrations of ASL *in vivo* generally uses secretions that are accessible (eg. nasal, bronchial) or techniques such as bronchoalveolar lavage (BAL; which significantly dilutes ASL) or exhaled breath condensate (of which only a small fraction is derived from ASL). *In vitro*, the depth of ASL generated by airway epithelial cells grown at air-liquid-interface remains ~7μm. Thus direct measurement of glucose in ASL remains challenging [4]. Nevertheless, using such techniques, the normal concentration of glucose in ASL (*in vitro* and *in vivo*) was measured as ~0.4 mM [5–8]. However, exposure to pro-inflammatory stimuli (e.g. in respiratory disease) or hyperglycaemia (e.g. diabetes mellitus) increased glucose concentration. When both are present, (e.g. Cystic Fibrosis-related diabetes) glucose concentration in the ASL was shown to reach ~4 mM [5]. This rise in glucose was associated with increased presence of respiratory pathogens such as methicillin-resistant *Staphylococcus aureus* and *Pseudomonas aeruginosa* [9,10]. The measurement of ASL glucose concentration *in situ*, particularly in the lower airways, and the measurement of dynamic changes in ASL glucose concentration in response to hyperglycaemic/pro-inflammatory challenge remain difficult because of the low volume and inaccessibility of samples. Measurement of glucose in ASL using fluorescent GBPs therefore provides a potential solution.

GBP alters its conformation when glucose binds and when linked to an environmentally-sensitive fluorophore it permits glucose binding to be detected as a change in fluorescence. GBP has previously been used as a core for several fluorescent glucose biosensors [11–13]. The original GBP has a K_d_ for D-glucose of approximately 0.8 μM [14]. Therefore, we introduced mutations H152C/A213R to decrease the affinity for D-glucose, in order to obtain a sensor with a K_d_ (~1mM) to be able to measure glucose concentration in ASL. The mutated GBP linked to the fluorophore BADAN gave the most useful *K*_d_ and the biggest dynamic range. It also had a clear specificity for D-glucose. However, the wavelength of BADAN was too low for effective *ex vivo/in vivo* imaging. Linking the H152C/A213R GBP to IANBD increased the *K*_d_ for D-glucose to an unusable 25 mM and longer wavelength fluorophores Blue oxazine, Nile Blue and Chromis 678 had very limited dynamic range.

We hypothesised that several other mutations could be utilised to obtain a GBP linked to a longer wavelength fluorophore, with an appropriate K_d_ and dynamic range for D-glucose. We have explored these mutations and identified a new candidate GBP which can be used to effectively measure glucose concentration in ASL overlying airway epithelial cells *in vitro*, the presence of raised glucose in *ex vivo* BALf and could be developed further for *in vivo* imaging.

## Materials and methods

### Point-mutation polymerase chain reaction (PCR)

C-terminal His tagged pET303-GBP plasmid was a kind gift of Dr Faaziah Khan (Kings College London). Site-directed mutagenesis primers were designed using QuickChange primer design online (Agilent Genomics) (Table 1). Site-directed mutagenesis primers (0.2 μM) were added to 100 ng of pET303-GBP template DNA, 2.5 units of DNA polymerase PfuUltra II, 0.8 mM dNTP, 17% DMSO in PfuUltra II buffer (Agilent technologies) and subjected to 18 cycle PCR with denaturation 95°C for 50 secs, annealing 60°C for 50 secs and elongation at 68°C for 7 mins. Methylated template DNA was digested with DpnI (20 units; New England Biolabs) overnight at 37°C. The DNA was transformed into XL10 *E*. *coli* and plasmids extracted using a using a QIAGEN HiSpeed Plasmid Midi kit (Qiagen, Venlo, Netherlands). Mutations were confirmed by sequencing (Beckman Coulter genomics).

**Table 1:** Site directed mutagenesis primers.

Desired Mutation	Forward primer (5’-3’)	Reverse primer (5’-3’)
**F16C**	ctataagtacgacgataactgtatgtctgtagtgcgcaagg	ccttgcgcactacagacatacagttatcgtcgtacttatag
**Q140C**	ttgggatctgaacaaagacggttgcattcagttcgtactgctgaaag	ctttcagcagtacgaactgaatgcaaccgtctttgttcagatcccaa
**E149C**	gattcagttcgtactgctgaaaggttgcccgggccatccgg	ccggatggcccgggcaacctttcagcagtacgaactgaatc
**V207C**	cgccaacaaaatcgaagtgtgtatcgccaacaacgatgcg	cgcatcgttgttggcgatacacacttcgattttgttggcg
**A213R**	tatcgccaacaacgataggatggcaatgggcgcg	atagcggttgttgctatcctaccgttacccgcgc
**L238S**	ttggcgtcgatgcgtcgccagaagcgctgg	ccagcgcttctggcgacgcatcgacgccaa

### GBP protein expression and purification

Plasmids were transformed into *E*. *coli* BL21 DE3 Gold (Agilent Technologies). Bacteria were grown at 37°C, 225 rpm in Lennox LB broth plus 50 μg/ml ampicillin until the OD_600nm_ was between 0.4–0.6. Protein expression was induced with 0.5 mM isopropyl-beta-D-thiogalactopyranoside (IPTG) overnight at 20°C. *E*. *coli* were then pelleted and lysed using 1 mg/ml lysozyme plus sonication. The sample was then centrifuged at 11,000 rpm, 4°C for 60 minutes and the supernatant applied to a nickel nitrilotriacetic acid (Ni-NTA) (Qiagen) column. The protein was eluted in 1.5 ml fractions. 15 μl of each fraction was subjected to SDS-PAGE analysis using 4–12% Bis-Tris gel (Life Technologies) to confirm the presence of the 35kDa GBP. Protein concentration was determined using a Nanodrop 1000 spectrophotometer (Thermo Scientific).

### Labelling proteins with fluorophores

Purified proteins were labelled with environmentally sensitive fluorophores (Table 2), either 6-Bromoacetyl-2-Dimethylaminonaphthalene (BADAN) (Thermo Fisher) or N,N’-Dimethyl-N-(iodoacetyl)-N’-(7-nitrobenz-2-oxa-1,3-diazol-4-yl)ethylenediamine (IANBD) (Life Technologies) by combining 100 μM protein, 50 μM tris(2-carboxyethyl)phosphine (TCEP), 500 μM fluorophore and PBS to a volume of 500 μl and incubating on ice overnight at 4°C. The protein was then dialysed in PBS using 12kDa dialysis pods (Spectrum Labs) overnight at 4°C. Proteins labelled with Nile blue or Chromis 678 utilised click chemistry. GBP-alkyne was obtained by incubating 100 μM of GBP with 10-fold excess of iodoacetamide alkyne in PBS, 2.5 mM TCEP, pH 7.4, for 2 h at room temperature. Excess of alkyne was removed by extensive dialysis in PBS pH 7.4 at 4°C (Slide-a-Lyser 10 kDa, Pierce). This was then incubated with the dye-azide using the Click-iT^®^ Protein Reaction Buffer kit for 1 h at room temperature. The excess of dye was removed by extensive dialysis as above. Dye protein conjugate concentrations are reported as protein concentration.

**Table 2:** Fluorophores attached to glucose binding protein mutants and their respective excitation and emission wavelengths.

Fluorophore	Excitation (nm)	Emission (nm)
**BADAN**	387	535
**IANBD**	480	536
**Nile blue azide**	625	670
**Chromis 678 azide**	677	701

### Equilibrium glucose binding

Equilibrium glucose binding curves were obtained by continuous titration followed by corrections for dilution and photobleaching. To determine the dynamic range (*F*_max_/*F*_0_), the cooperativity (*n*) and the dissociation constant for D-glucose (*K*_d_) of the labelled GBP proteins, glucose affinity assays were performed using an automated syringe pump (ALADDIN 1000, WPI). Labelled GBP proteins (40–100 nM) were titrated continuously with Glucose at 10–20 μL/min flow rate in a stirred 3 mL cuvette, in PBS pH 7.4. Fluorescence was measured at fluorophore excitation and emission wavelength peaks (Table 2) at 20°C on a Fluorolog3 spectrofluorimeter (Horiba Scientific). Data were expressed as the mean of triplicates ± SEM. The fluorescence changes were normalised and fitted to the Hill equation Y = Bmax*X^*n*/(Kd^*n* + X^*n*) using Prism GraphPad 6 software. As GBP/glucose buinding should not exhibit co-operativity *h* was constrained to ≤1.

### Airway epithelial cells

H441 airway epithelial cell line was obtained from ATCC. Human bronchiolar epithelial cells HBECs were obtained by the UNC Marsico Tissue Culture Core from endobronchial brushings or extracted from explanted normal lungs as previously described. Human Subject Research Ethical approvals were granted by The University of North Carolina at Chapel Hill Biomedical Institutional Review Board (protocol #03–1396) [22]. H441 and HBEC were transferred onto transwell permeable supports (Costar) and grown at air-liquid-interface (ALI) to form confluent monolayers as previously described [10]. For studies of glucose in airway surface liquid (ASL), cells were transferred into basolateral HEPES buffered Kreb’s solution (HEPES 24 mM, NaCl 101 mM, NaHCO_3_ 12 mM, MgCl_2_ 1.2 mM, CaCl_2_.2H_2_O 1.2 mM, KCl 5.2 mM) with either 5 mM D-glucose + 10 mM L glucose or 15 mM D-glucose.

### ASL fluorescence analysis

E149C/A213R GBP-IANBD (20μl of 0.3μM) or vehicle control was added to the apical surface of H441 or HBEC grown at ALI with known concentrations of D-glucose (to obtain a dose response) or without glucose (to estimate glucose concentration in the ASL) and allowed to equilibrate for 10 minutes at 37°C in a humidified incubator. Fluorescent output was read by a TECAN infinite F200 PRO plate reader excitation/emission 485/535nm. The fluorescence emission of IANBD or vehicle control was analysed from 9 points within the Transwell (points were taken a minimum of 7.5mm from the edge of the well). Settings were optimised differently for H441 and HBEC due to the differences in monolayer thickness and ASL heights in these cell types. Vehicle control background values were subtracted from sensor fluorescent outputs to obtain values for E149C/A213R GBP-IANBD fluorescence. For time course studies emission intensity was collected every minute for the first 10 minutes, followed by readings every 10 minutes up to an hour, then every hour until 5 hours had elapsed. Alternatively, E149C/A213R GBP-IANBD was added together with 20μl 0.5 mg/ml of 10 kDa dextran-tetramethylrhodamine (TMR; Life Technologies, USA) as a labelling control for the ASL. Cells were labelled with nuclear stain Hoechst 33342. Images were acquired in XZ-scanning mode by using a Leica SP8 confocal microscope with a ×63/1.3 numerical aperture (NA) glycerol immersion lens. Cells were exposed to either 5mM D- + 10mM L-glucose or 15mM D- glucose basolaterally for 10 minutes before imaging. Image intensities were analysed using ImageJ software (NIH).

### Bronchoalveolar lavage glucose measurements

9-week-old C57B/6 and BKS.Cg-m+/+Lepr^db^/J (db/db) (6 of each group) mice were terminated with a single lethal intraperitoneal injection of 0.2 mL pentobarbital (60 mg/ml) diluted 1:1 with saline. Prior to termination, blood was collected from the vena saphena of the hindlimb, and blood glucose was measured with an Accu-Chek^®^ Mobile meter (Roche). The trachea was exposed and a catheter inserted between two cartilage rings. The catheter was secured with a silk suture and the lungs were subjected to manual lavage with 2 x 0.8 ml PBS. Retrieved BALf samples were centrifuged at 1200 rpm, 10 min, at 4°C and the supernatant stored at -80°C until use. Bronchoalveolar lavage samples were subject to glucose measurement by E149C/A213R GBP-IANBD or Amplex red glucose oxidase assay (Thermo Fisher). Standard curves were generated by adding known glucose concentration (from 0–10 mM) to 50 μl of either 0.3μM E149C/A213R GBP-IANBD or Amplex red reaction mix. Fluorescence output was measured on a GloMax plate reader, excitation/emission 590/536 nm or 530/480 nm for E149C/A213R GBP-IANBD or Amplex red respectively.

For *in vivo* studies, mice (as above) were anaesthetised in Isoflurane 4–5% (O_2_ 1.2 L/min), held vertically and 50 μl of 0.3 μM E149C/A213R GBP-IANBD, 50 μl of positive control (0.3 μM E149C/A213R GBP-IANBD pre-mixed with 10 mM D-glucose), or 50 μl of PBS vehicle control were gently dropped by pipette onto one nostril for inhalation. Five minutes post inhalation, mice were sacrificed as above and lungs were excised, frozen in optimal cutting temperature compound (OCT) and 7 μm cryosections obtained. Images were then obtained using a Nikon A1R confocal microscope using a 20x CFI plan fluor lens. As there is significant auto fluorescence in lung tissue, spectra were acquired from the same emission window for IANBD and lung tissue alone. The spectra associated with IANBD and autoflourescence were then separated and artificially coloured on the acquired images (spectral un-mixing). Intensities were measured with ImageJ in regions surrounding the lumen for both the C57B/6 and db/db mice. Animal Research Ethical approval was granted by Ethical committee in Gothenburg, Sweden (184–2012).

## Results

As the wild-type *E*. *coli* GBP sequence does not contain a cysteine residue (C), single cysteine substitutions were firstly made to enable thiol bonds to be formed with BADAN and the IANBD fluorophore. Our aim was to produce a sensor with a K_d_ for glucose of ~1 mM and the greatest dynamic range (F_max_/F_0)_ possible.

### Single amino acid substitutions to GBP labelled with BADAN

**Fig 1:**
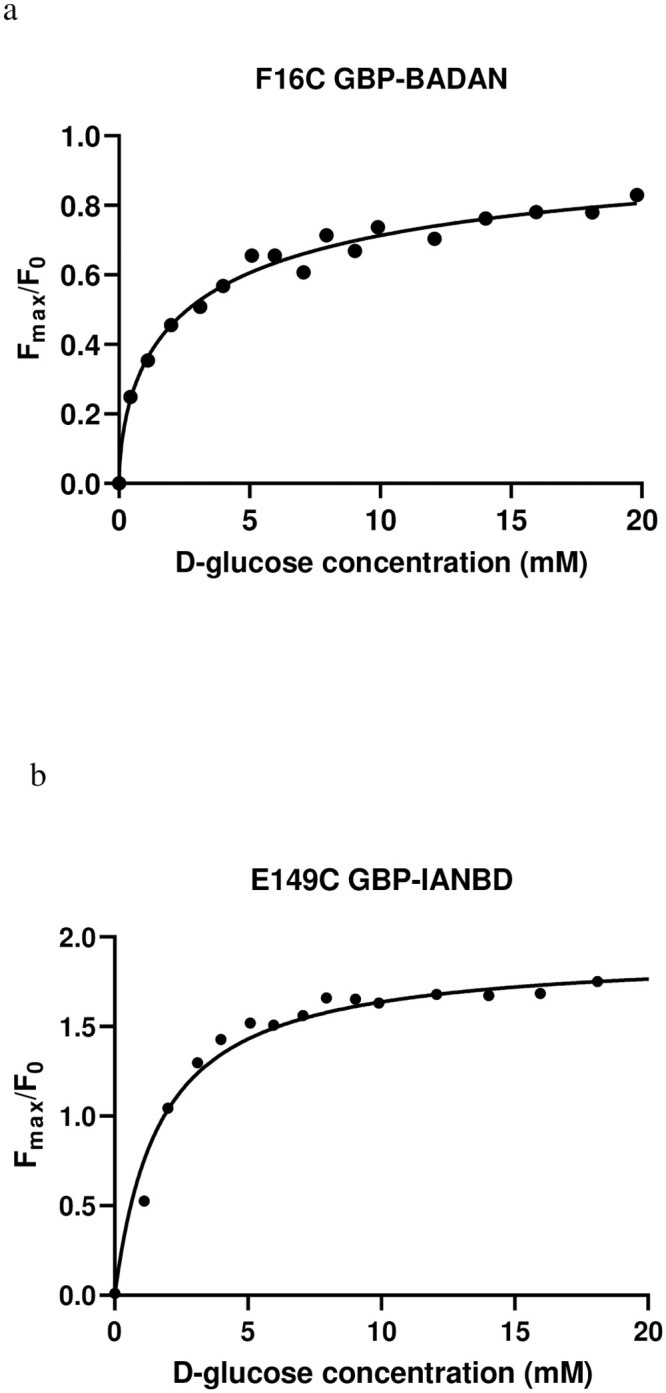
Fmax/F0 D-glucose concentration response curves for two single point GBP mutations. a. F16C labelled with BADAN and b. E149C labelled with IANBD. Curves were fitted using the one site-specific binding with Hill slope formulae in GraphPad prism.

Phenylalanine at position 16 (F16) is located within the glucose binding pocket of GBP. The change in protein conformation upon glucose binding exposes the F16 residue to less solvent. Thus, it was anticipated that this would induce a change in fluorescence. As predicted, GBP F16C-BADAN had a calculated K_d_ to D-glucose of 3.2 mM (R^2^ = 0.98) but the fluorescence dynamic range was small F_max_/F_0_ of 0.83 (Table 3, Fig 1a). The glutamine at position 140 (Q140) is located on one of the two lobes of GBP. Substitution with cysteine (Q140C) resulted in no change in fluorescence emission with the addition of glucose and we were unable to calculate a K_d_. (Table 3). Glutamic acid at position 149 (E149) is located on the lip of the GBP binding cleft. Previous studies had shown good fluorescence dynamic range for sensors with a fluorophore attached to this residue [15,16]. E149C produced the largest fluorescent range of all the single mutation sensors bound to BADAN with an F_max_/F_0_ of 0.97, and the K_d_ of the sensor to D-glucose was too high at 84.2 mM (R^2^ = 0.98) (Table 3). The valine amino acid at position 207 is located within one of the lobes of the GBP. We were unable to gain a K_d_ and dynamic range data for V207C GBP-BADAN (Table 3).

**Table 3:** Summary of the biochemical characteristics of GBP-labelled mutants.

Protein	Fluorophore	K_d_ (mM)	R^2^	F_max_/F_o_
F16C	BADAN	3.2	0.98	0.83
Q140C	BADAN	n/o	n/o	n/o
E149C	BADAN	84.2	n/o	0.97
V207C	BADAN	n/o	n/o	n/o
F16C	IANBD	3.2	0.98	0.124
Q140C	IANBD	2.03	0.96	0.06
E149C	IANBD	2.01	0.97	1.96
V207C	IANBD	n/o	n/o	n/o
E149C L238S	BADAN	1.6	0.93	0.98
E149C A213R	BADAN	11.2	0.98	0.88
E149C L238S	IANBD	9.36	0.97	1.21
E149C A213R	IANBD	1.01	0.91	5.8
E149C A213R	Chromis 678	>190	0.90	n/o
E149C A213R	Nile Blue	n/o	n/o	n/o

Not obtained (n/o).

### Single GBP substitutions labelled with IANBD

Each of the single mutant proteins was then labelled with the environmentally sensitive fluorophore IANBD and the fluorescence in response to glucose measured. Of the four, the F16C GBP mutant exhibited only a small increase in fluorescence with glucose binding with an F_max_/F_0_ of 0.124, whereas the E149C GBP produced the largest change in fluorescence with an F_max_/F_0_ of 1.96, and a K_d_ to D-glucose of 2.01 mM (R^2^ = 0.98) (Table 3, Fig 1). Both the Q140C and V207C mutants displayed negligible increases in fluorescence upon addition of glucose with F_max_/F_0_ of 0.06 and 0.01 respectively (Table 3). As E149C produced the best Kd and F_max_/F_0_ for D-glucose with both fluorophores we then introduced further amino acid substitutions with an aim to increase the dynamic range (F_max_/F_0_).

### Dual amino acid substitutions to GBP labelled with BADAN

**Fig 2:**
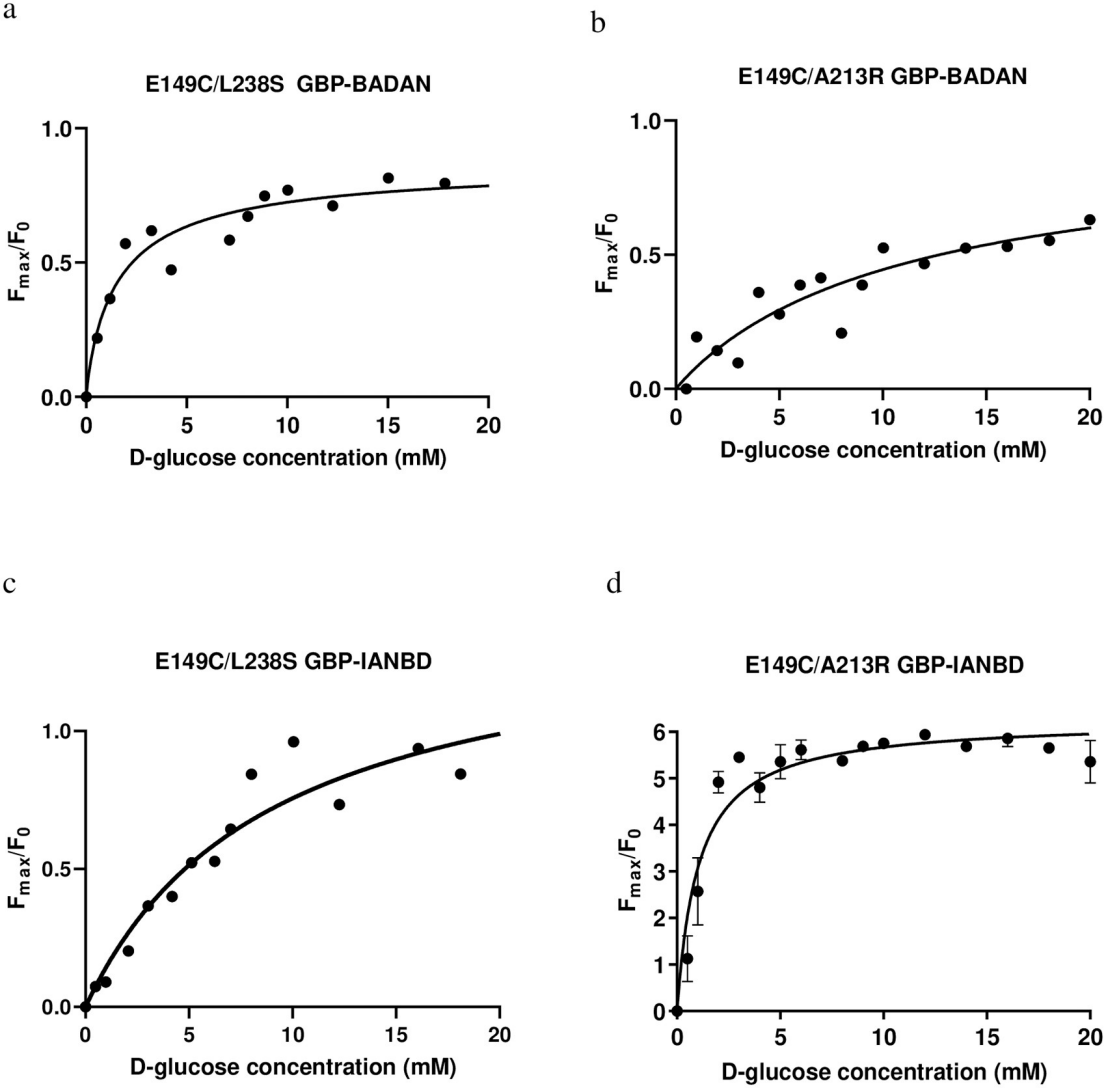
Fmax/F0 D-glucose concentration response curves for each of the dual mutation GBPs. a. E149C/L238S and b. E149C/A213R labelled with BADAN, c. E149C/L238S and d. E149C/A213R labelled with IANBD. Curves were fitted using the one site-specific binding with Hill slope formulae in GraphPad prism.

Leucine (L238) is within close proximity to E149, (14.5Å in its open conformation and 12.8Å in its closed conformation). Although L238 is located near the binding cleft of the protein, it makes no polar contact with the glucose molecule. Substituting leucine for serine (S) created a double mutant E149C/L238S GBP-BADAN, which exhibited a K_d_ to D-glucose of 1.6 mM (R^2^ = 0.93) but the dynamic range remained small F_max_/F_0_ of 0.98 (Table 3, Fig 2a). Alanine (A213) is also located in the same region in close proximity to E149 (11.8Å when in the open conformation and 9.3Å apart when in the closed conformation) and located near the binding cleft [17]. A213 was substituted with arginine (R). This has a charged side chain which we predicted would result in a greater shift in the polarity of the environment surrounding the fluorophore (See supporting information, S1 Fig). The double mutant E149C/A213R GBP-BADAN had a K_d_ for D-glucose of 11.2 mM (R^2^ = 0.94) and an F_max_/F_0_ of 0.88 (Table 3, Fig 2b).

### Dual amino acid substitutions to GBP labelled with IANBD

The dual mutant E149C/L238 GBP-IANBD had a calculated K_d_ for D-glucose of 9.36 mM (R^2^ = 0.97) and F_max_/F_0_ of 1.21. Interestingly, the E149C/A213R GBP-IANBD K_d_ to D-glucose of 1.02 mM (R^2^ = 0.91; n = 2) and an F_max_/F_0_ of 5.8 (Table 3, Fig 2c and 2d).

As the E149C/A213R GBP sensor produced the largest F_max_/F_0_ when labelled with IANBD and had a K_d_ for D-glucose in the concentration range ideal for measuring ASL glucose, the protein was selected to label with longer wavelength fluorophores for potential future *in vivo* applications.

### Dual amino acid substitutions to GBP labelled with Chromis 678 or Nile Blue

**Fig 3:**
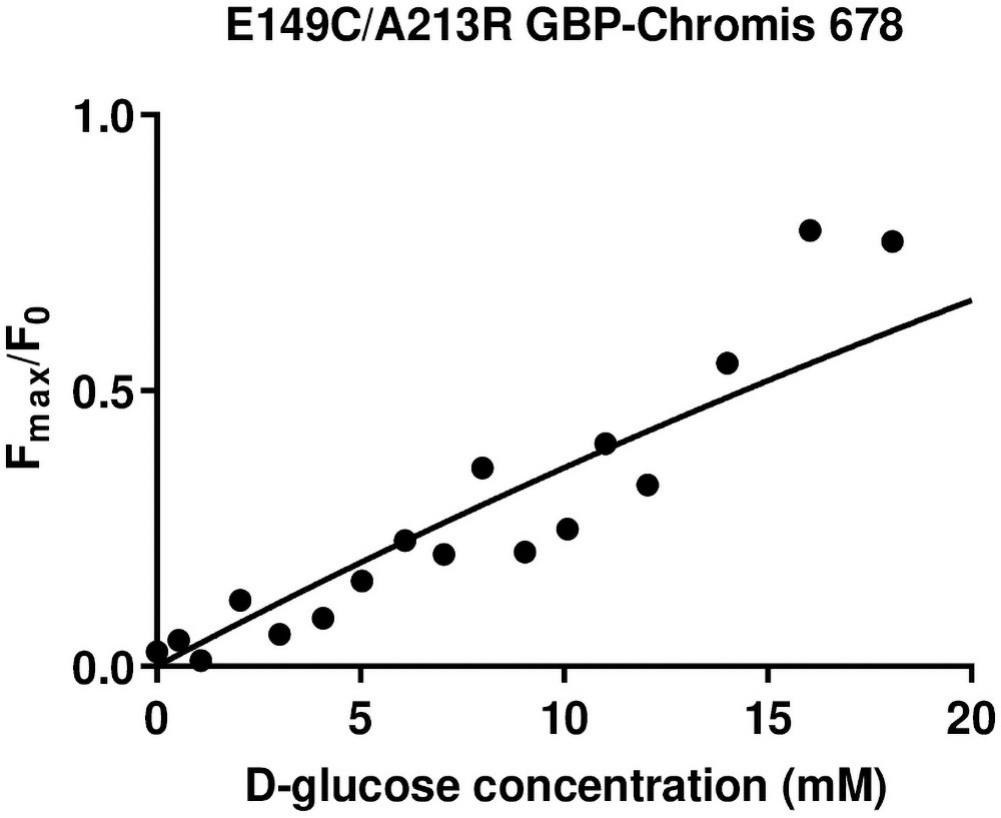
Fmax/F0 D-glucose concentration response curve for the dual mutant E149C/A213R GBP labelled with Chromis 678. The curve was fitted using the one site-specific binding with Hill slope formulae in GraphPad prism.

E149C/A213R GBP labelled with Chromis 678 produced a sensor with a K_d_ for D-glucose of 190 mM (R^2^ = 0.9) and an F_max_/F_0_ of 1.43 over the glucose concentration range tested. This was a smaller fluorescence range and larger K_d_ than when the protein was labelled with IANBD. Attaching Nile blue to the mutant GBP resulted in no glucose-dependent fluorescence (Table 3, Fig 3).

### Selectivity of E149C/A231R GBP-IANBD for D-glucose

**Fig 4:**
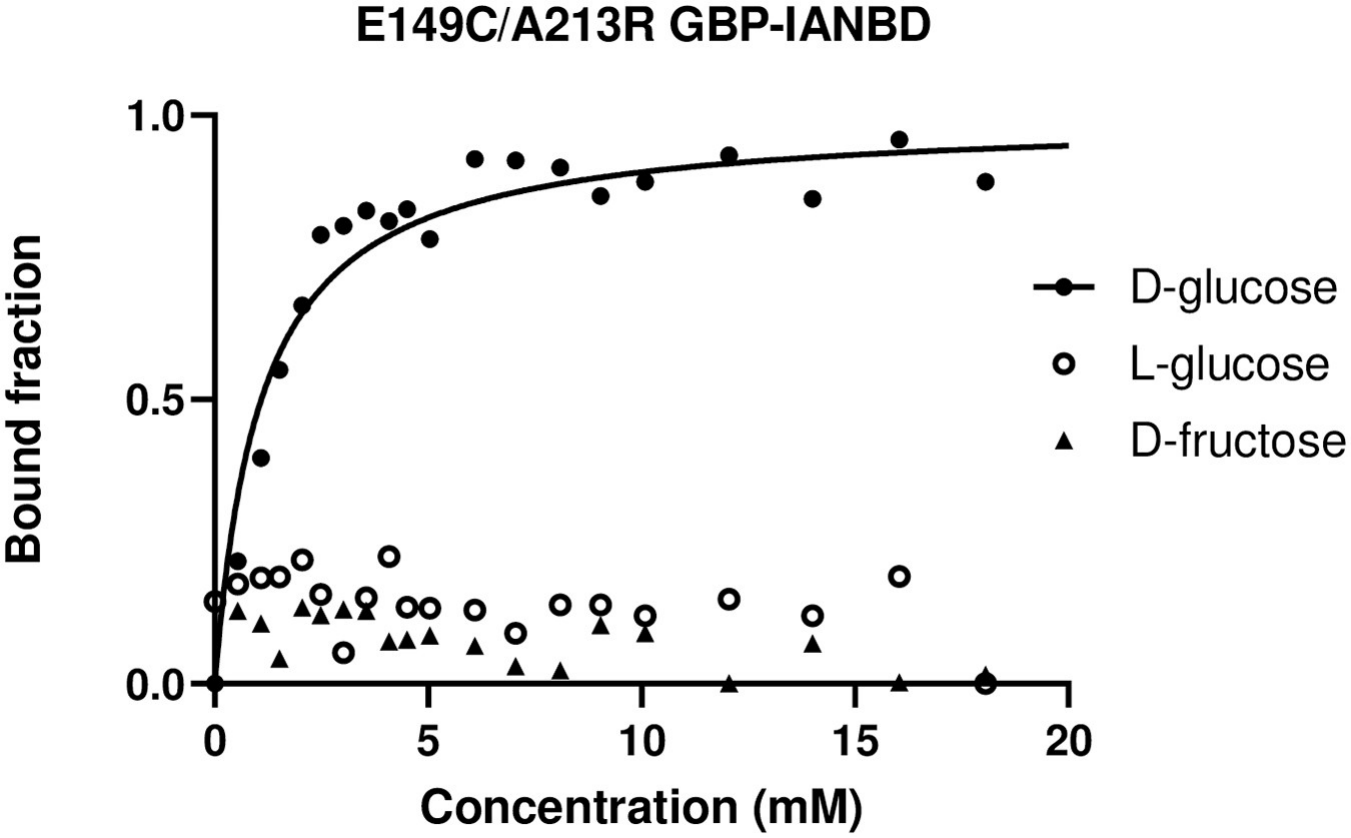
Fluorescence associated with bound fraction of E149C/A213R GBP-IANBD with increased concentration of D-glucose (circles) compared to L-Glucose (squares) and D-fructose (triangles). The curve for D-glucose was fitted using the one site-specific binding with Hill slope formulae in GraphPad prism. Curves could not be fitted for L-glucose or D-fructose.

As labelling with the longer wavelength fluorophores resulted in a decrease in fluorescence range, the best sensor we produced was E149C/A213R GBP-IANBD. Introducing mutations can have an effect on the specificity of the protein to sugars. Therefore, we investigated changes in fluorescence in the presence of L-glucose (enantiomer of D-glucose) and D-fructose which is found in small quantities in the ASL. In concentrations up to 20 mM (a concentration which is significantly higher than that found in the airways), the addition of L-glucose or D-fructose to the E149C/A213R GBP-IANBD sensor did not result in a change in fluorescence, indicating that the protein was selective for D-glucose (Fig 4).

### *In vitro* application of E149C/A213R GBP- IANBD

**Fig 5:**
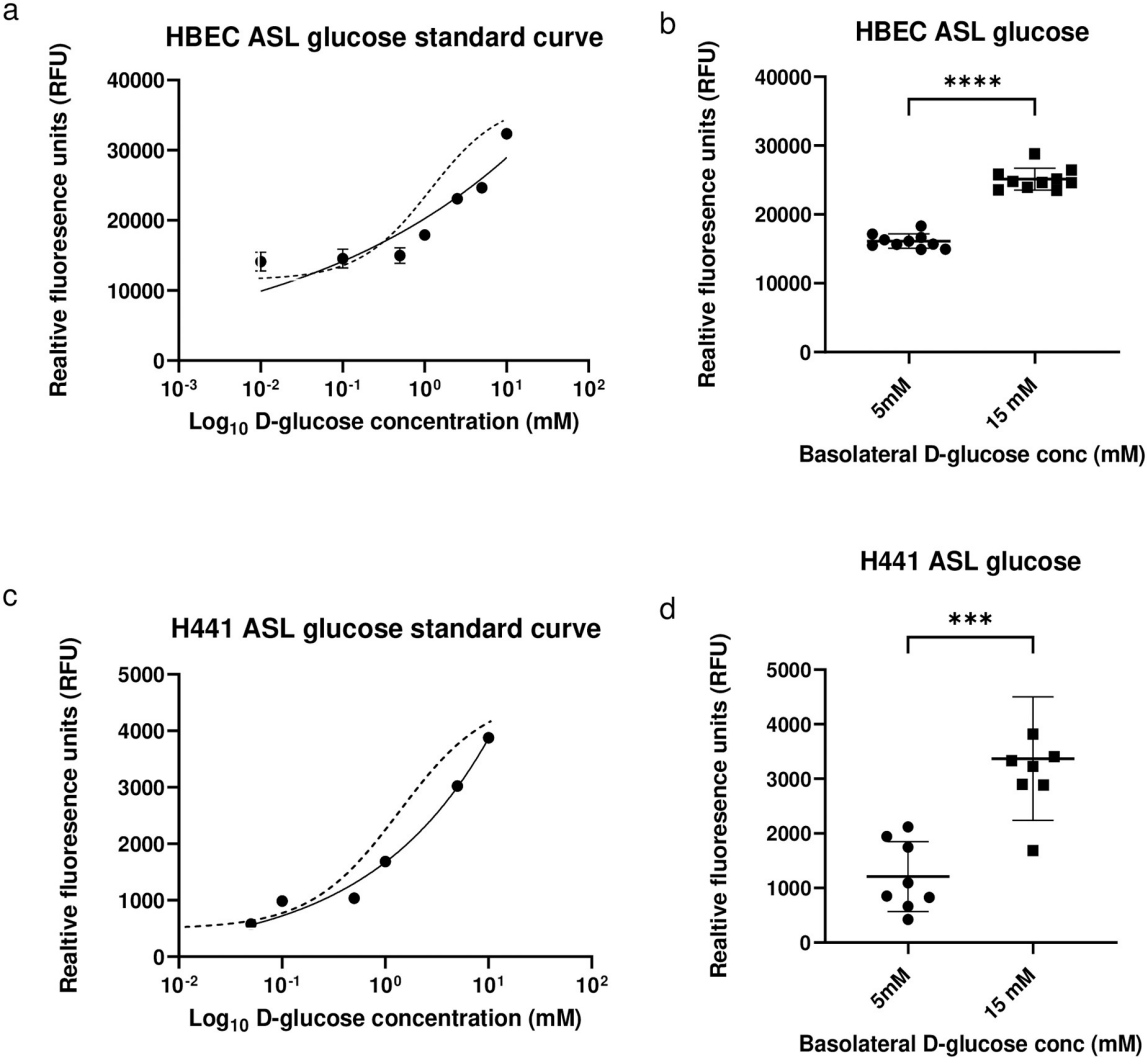
Fluorescence of E149C/A213R GBP-IANBD applied to the ASL of HBEC or H441 grown at air-liquid-interface together with known concentrations of glucose to obtain a concentration effect curve a. and c. The response curve measured in PBS over the same glucose concentration range is shown as a dotted line in both graphs. Fluorescence of E149C/A213R GBP-IANBD applied to the ASL of HBEC or H441 without added glucose to measure changes in ASL glucose when basolateral glucose concentration was raised from 5mM to 15 mM D-glucose in the basolateral chamber for 10 minutes b. and d. Data are shown as mean ± SD. Significantly different ** p<0.001, **** p<0.0001.

We took two approaches *in vitro*. First, we applied the sensor to the apical/luminal surface of H441 and HBEC airway calls grown at air-liquid interface to see if we could detect changes in the airway surface liquid (ASL) glucose concentration with a hyperglycaemic challenge. We measured fluorescence of E149C/A213R GBP-IANBD when applied with known concentrations of D-glucose to the ASL of HBEC and H441 grown on transwells. Settings were optimised differently for HBEC and H441 due to the differences in monolayer thickness and ASL heights in these cell types. Fluorescence increased with glucose concentration in the ASL (Fig 5a and 5c). However, over the same concentration range the change in IANBD fluorescence in both cases appeared reduced when compared to that measured in PBS. Then we applied E149C/A213R GBP-IANBD without added glucose to measure fluorescence in the ASL when the D-glucose concentration of the basolateral/serosal bath was increased from 5 to 15 mM. Fluorescence increased by from 16126 ± 1045 to 25138 ± 1593 relative fluorescence units (RFU) (p≤ 0.0001; n = 10) in HBEC cells and from 1209 ± 639 to 3370 ± 1132 RFU (p≤ 0.001; n = 8) in H441 respectively (Fig 5b & 5d). Resolving the mean change in fluorescence using the concentration effect curves indicated an estimated change in glucose concentration in the ASL from 0.2±0.1 to 4.3±2.1 mM in HBEC and 0.7 ± 0.7 to 5.8 ± 2.5 mM in H441 cells when basolateral glucose was increased from 5–15 mM (p<0.0001, n = 10 and 8 respectively).

**Fig 6:**
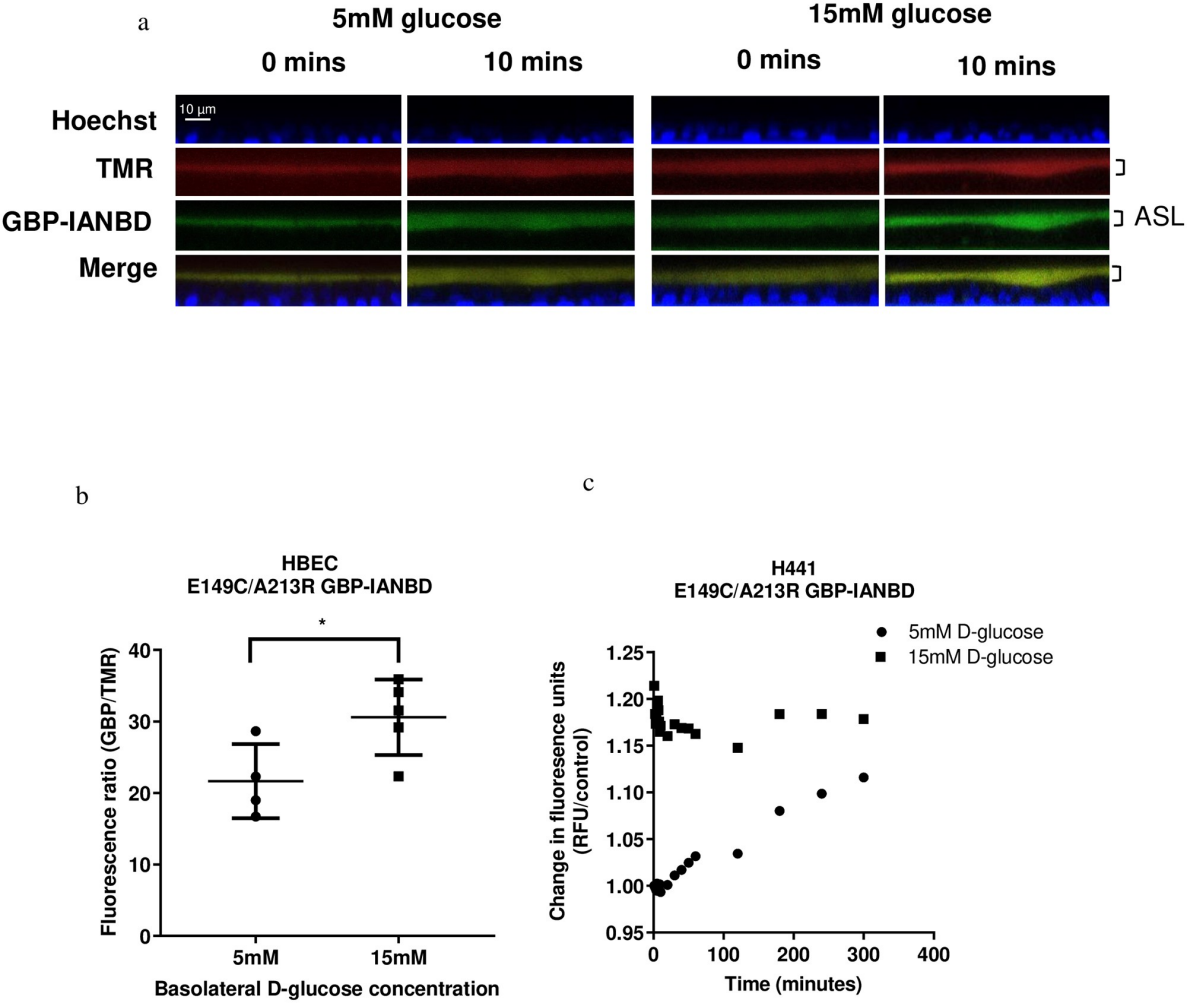
Exemplar confocal XZ images of HBEC cells grown at air-liquid interface stained with Hoechst (blue) to label nuclei, dextran tetramethylrhodamine (TMR, red) to label airway surface liquid (ASL) and E149C/A213R GBP-IANBD (GBP-IANBD, green) in ASL. A merged image (Merge) in which the overlay of TMR and GBP-IANBD appears as yellow. Images were collected from cells exposed to 5 mM or after raising to 15 mM D-glucose in the basolateral chamber for 10 minutes. b. Collated confocal data of ASL fluorescence ratio (GBP-IANBD/TMR) for HBEC shown in a. Data are shown as mean ± SD. Significantly different * p<0.05. c. Change in fluorescence of E149C/A213R GBP-IANBD applied to the apical surface of H441 grown at air-liquid-interface with either 5 mM or after raising to 15 mM D-glucose in the basolateral chamber for up to 300 minutes measured by fluorimeter. A size bar (10μm) applicable to all images is shown in the top left-hand image.

Second, we investigated the potential of E149C/A213R GBP-IANBD for observing changes in glucose concentration in the ASL by microscopic imaging. Confocal X-Z images of the cells clearly captured IANBD and dextran-tetramethylrhodamine (TMR) fluorescence in the ASL overlying the epithelial cells (Fig 6a). The fluorescence of E149C/A213R GBP-IANBD but not TMR was increased when basolateral D-glucose was raised from 5 to 15 mM. IANBD/TMR fluorescence intensity ratio increased from 21.7±5.2 (n = 4) to 30.6±5.3 (n = 5) (p≤ 0.05) at 10 minutes (Fig 6b). Taken together, these data indicate that increasing basolateral glucose increased glucose in the ASL which is sensed by E149C/A213R GBP-IANBD. Finally, using the fluorimeter protocol, we analysed the fluorescence stability of E149C/A213R GBP-IANBD applied to the apical surface of H441 grown at air-liquid-interface with either 5 mM or after raising to 15 mM D-glucose in the basolateral chamber for up to 300 minutes. Fluorescence did not decline over time and remained relatively stable in cells exposed to 15mM glucose. It increased in cells exposed to 5mM glucose but values did not converge (Fig 6c).

### Bronchoalveolar lavage fluid (BALf)

**Fig 7:**
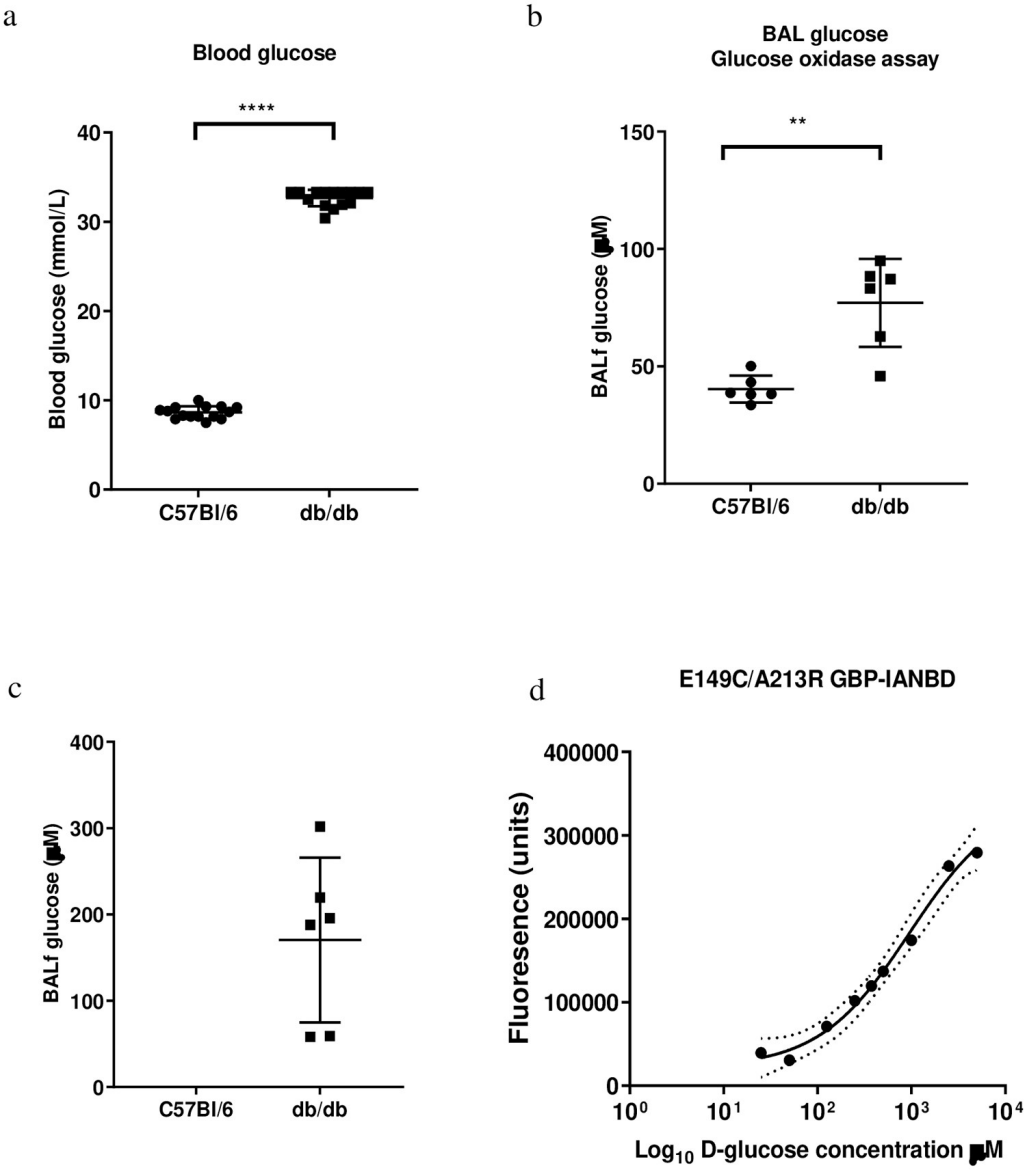
a. Blood glucose concentration (mmol/L), Bronchoalveolar lavage (BAL) glucose concentration measured by b. glucose oxidase assay and c. E149C/A213R GBP-IANBD for C57Bl/6 and db/db mice used in study. Data are shown as mean ± SD. Significantly different ** p<0.01, **** p<0.0001. d. E149C/A213R GBP-IANBD fluorescence/D-glucose concentration curve. Data were fitted with a curve using Graphpad prism.

After confirmation that the sensor increased fluorescent output in response to glucose *in vitro*, application of the sensor was progressed into murine models of normoglycaemia (wildtype C57Bl/6) and hyperglycaemia (db/db). Blood glucose was higher in db/db (32.65 ± 0.93 mmol/L) compared to C57Bl/6 (8.64 ± 0.68 mmol/L) (p≤ 0.0001; n = 12) (Fig 7a). For 9 of the db/db mice, blood glucose exceeded the limit of detection of the Accu-Chek^®^ set at 33.3 mmol/L. We collected bronchoalveolar lavage fluid (BALf) from these mice (which contains diluted ASL). Glucose concentration in BALf by Amplex^®^ red glucose oxidase assay was 40.3 ± 5.7 μM and 77.1 ± 18.8 μM (p≤ 0.01; n = 6) for the wildtype and hyperglycaemic db/db mice respectively (Fig 7b). The glucose concentration using E149C/A213R GBP-IANBD in db/db mice was 170.4 ± 95.4 μM (calculated using a dose response curve for the glucose sensor in PBS) (Fig 7c & 7d). We were unable to detect changes in fluorescence in BALfs from C57Bl/6 mice and thus unable to calculate glucose concentration.

### Direct application of the sensor to the ASL *in vivo*

**Fig 8:**
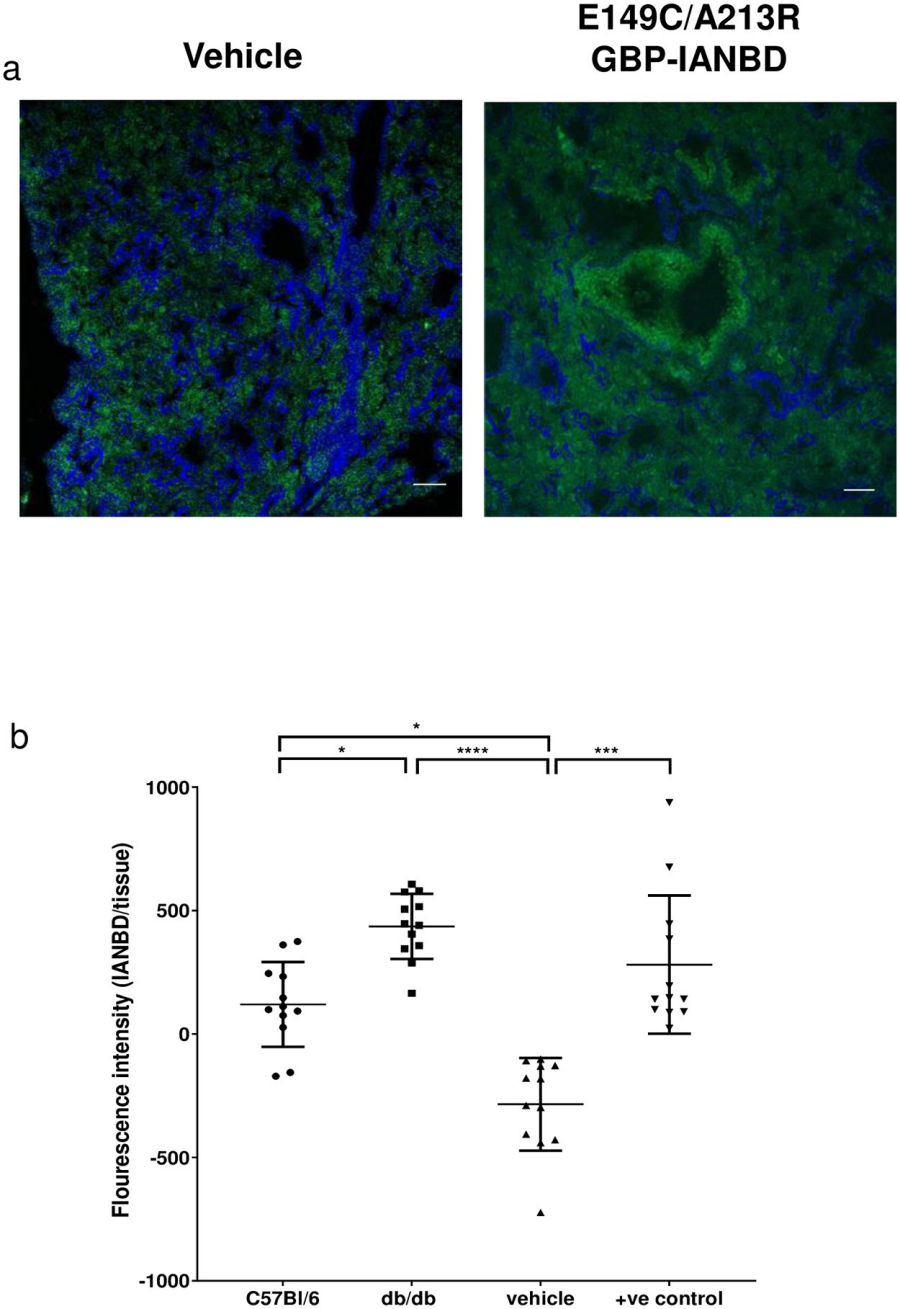
Exemplar images showing spectral un-mixing of cryo-sectioned C57Bl/6 (wild type) lungs treated with a. vehicle and b. E149C/A213R GBP-IANBD. Artificially coloured IANBD emission spectra collected from the sample (green), and artificially coloured lung tissue spectra (blue). Scale bar is 20 μm. Fluorescence intensity of E149C/A213R GBP-IANBD spectra surrounding the luminal regions were measured with ImageJ. c. collated data of fluorescence intensity of cryo-sections from C57Bl/6 or db/db mice treated with E149C/A213R GBP-IANBD, or C57Bl/6 treated with vehicle, or C57Bl/6 treated with E149C/A213R GBP-IANBD pre-bound with 10 mM D-glucose (+ve control). Data are shown as mean ± SD. Significantly different * p<0.05, *** p<0.001, **** p<0.0001.

As BALf contains highly diluted ASL, we investigated whether we could detect changes in glucose with E149C/A213R GBP-IANBD applied directly to the lung lumen. Unfortunately, the wavelength of IANBD has limited tissue penetration for direct *in vivo* imaging using some methodologies although two photon imaging is a possibility. Therefore, we tried to see if we could detect changes in luminal fluorescence in cryosections of lungs after mice had inhaled E149C/A213R GBP-IANBD. The lung has significant innate fluorescence so we used spectral un-mixing to separate IANBD fluorescence from tissue auto-fluorescence and the images were coloured accordingly (Fig 8a). E149C/A213R GBP-IANBD fluorescence was greater in hyperglycaemic db/db mice with a mean intensity of 436.0 ± 131.9 units (n = 12) than in normoglycaemic C57B/6 mice 119.9 ± 171.9 units (n = 12) (p≤0.05). Both C57B/6 and db/db mice had a higher E149C/A213R GBP-IANBD fluorescence intensity than the vehicle control (-284.6 ± 187.7 units, n = 12; p≤0.05 and p≤0.0001, respectively). Neither the db/db or C57B/6 intensities differed from the positive control of the sensor pre-bound with D-glucose prior to inhalation (Fig 8b). It was not possible to determine actual glucose concentration from this fluorescence data because we were unable to construct an appropriate dose response curve.

## Discussion

We previously generated a sensor H152C/A213R GBP-BADAN with a K_d_ for D-glucose of ~1 mM to measure glucose concentration in airway surface liquid (ASL) [18]. However, this was not suitable for work *in vivo*. Our aim was therefore, to further modify GBP and obtain a better sensor for this purpose. Several new mutations to GBP were investigated. Of these, the F16C GBP-BADAN gave the best readouts with a K_d_ to D-glucose of 3.2 mM and a respectable fluorescence dynamic range (F_max_/F_0_) of 0.83. However, E149C GBP was more useful for the longer wavelength fluorophore IANBD with a K_d_ to D-glucose of 2.01 mM and an F_max_/F_0_ of 1.96. These data are consistent with previous work from ourselves and others which show that the efficacy of the mutation in the GBP is not only dependent on its position relative to the glucose binding site but also how it interacts with the fluorophore and the solvent environment surrounding it [18,19].

Further mutations identified E149C/A213R GBP-IANBD with a K_d_ for D-glucose of 1.02 mM and a fluorescence dynamic range of 5.8. The success of this candidate most likely lay in the positions and properties of the two mutations. E149C, as it is close to the binding site, would likely affect the affinity for D-glucose, and A213R introduces a charged amino acid in close proximity to where the environmentally sensitive fluorophore is bound (within 11.8 Å when not bound to glucose and 9.3 Å when bound), altering the polarity of the environment and thus the fluorescent output (See supporting information, S1 Fig).

The longest wavelength fluorophore we could obtain, that worked with GBP for effective glucose sensing was IANBD with an emission wavelength maximum of 536 nm. This sensor was specific for D-glucose and exhibited fluorescence stability in experiments lasting up to 6 hours. Ideally, for a broadly applicable and effective *in vivo* sensor, the emission wavelength needs to be 650 nm– 900 nm to penetrate tissue as these wavelengths are minimally absorbed by haemoglobin and water [20,21]. The fluorophores Chromis 678 and Nile blue have emission maxima that sit within this window (Em_max_ 701 nm and 670 nm respectively). Binding of Chromis 678 to E149C/A213R GBP resulted in a very large increase in the K_d_ for D-glucose (<190 mM), indicating a significant hindrance of glucose binding to GBP. A similar effect of Chromis 678 was observed when bound to H152C/A213R GBP [18] and the H152 residue lies in close proximity to the E149 residue used in this study. Chromis 678 is larger than IANBD and has 4 benzene rings as opposed to one in IANBD. Thus, its size/structure likely impedes the glucose biding site. Nile blue bound to E149C/A213R GBP did not exhibit a change in fluorescence in response to glucose. Nile blue can produce strong dipole moments depending on the polarity of its surroundings making it a good environmentally sensitive dye [22]. Whilst Nile Blue had been shown to exhibit a change in fluorescence when bound to a different GBP mutant, this and our previous data indicate that long wavelength fluorophores bound to GBP consistently exhibit F_max_/F_0_ that currently are too low to be of practical use in the measurement of ASL glucose [18,23]. The use of quantitative structure activity relationship (QSAR) and docking study simulations of these mutants could help us design better long wavelength GBP sensors in the future.

*In vitro*, it has been shown that exposure of H441 cells and HBEC exhibited a rise in glucose on the apical surface when basolateral glucose was increased [9,19]. Measurement of glucose concentration in ASL is challenging because ASL volume is small and dynamically regulated by airway cells. We used L-glucose to control for the osmotic effects of raising D-glucose concentration. We also used TMR as a volume marker in confocal experiments and for ratio metric analysis with E149C/A213R GBP-IANBD because we previously showed that the fluorescence of TMR did not change in response to glucose [18]. We chose a consistent concentration of E149C/A213R GBP-IANBD and TMR to achieve the best fluorescent output but recognise there are limitations associated with potential concentration dependence differences in fluorescent output of IANBD and TMR.

Nevertheless, the use of E149C/A213R GBP-IANBD in the ASL showed an increase in IANBD fluorescence compared to TMR 10 minutes after raising basolateral glucose. This was more rapid than that previously reported for a BADAN labelled GBP, which conveyed changes after a two hour exposure [18]. In addition, fluorescent analysis of XZ sections by confocal microscopy using this ratio metric method gave similar outcomes and similar changes in relative fluorescence to that of fluorometric analysis of E149C/A213R GBP-IANBD applied alone to the ASL when basolateral glucose was raised from 5 to 15 mM. As E149C/A213R GBP-IANBD is specific, rapid and stable we suggest it could be a better sensor for interrogating epithelial regulation of ASL glucose.

In support of this notion, the sensor revealed a difference in the fluorescence emission in response to increased basolateral glucose in H441 and HBEC. There was a bigger change in H441 cells than HBEC and the variability of the HBEC data was smaller than that of H441. Using known glucose concentrations applied to the ASL we constructed dose response curves. Resolving the glucose concentrations for the ASL showed a similar trend between H441 and HBEC and values were consistent with previous findings [9,24,25]. These differences are likely related to the typically lower TEER of H441 compared to HBEC (~300 Ωcm^2^ and ~800 Ωcm^2^, respectively) and thus a potentially higher transepithelial permeability to glucose of H441. Differences in glucose transporters and their distribution (GLUT2 and 10 in H441 and GLUT1,2,4 and 10 in HBEC), uptake and metabolism between the cell types could also result in tighter control of glucose movement into and out of the ASL by HBEC [6,19,24,26–29]. Interestingly the dose response curves in ASL were slightly shifted to the right compared to those measured in PBS indicating that factors in ASL might temper sensor fluorescence. ASL pH of 7.1–7.2 was not altered in NHBE when exposed to elevated glucose, binding of acrylodan bound GBP was stable in the pH range 6.5–8.0 [30] and IANBD is stable at pH7.0. However, ASL viscosity could influence calibrations and this requires further investigation.

When used to measure glucose concentration in BALf, there were limits in the sensitivity of the sensor. Consistent with previous findings, db/db mice had higher blood glucose and more glucose in their lungs compared to wildtype mice as measured by glucose oxidase assay and fluorescence emission from E149C/A213R GBP-IANBD [31]. However, no fluorescence above baseline was detected with the sensor in wildtype BALf indicating a lack of glucose. During BAL the constituents of the ASL, including glucose, are significantly diluted. Very low concentrations of glucose can be detected in enzymatic assays which amplify the signal in a non-reversible end point reaction. The GBP sensor, on the other hand, reacts in a reversible 1:1 ratio with glucose, and this may limit its sensitivity in comparison. In this study, Amplex red assay glucose concentration in the BALf of wildtype mice was 33 μM. At this concentration, the change E149C/A213R GBP-IANBD fluorescence would be negligible, and thus, the sensitivity of the sensor may be limited in this scenario.

*In vivo* imaging in lungs is challenging. As a proof of concept we delivered E149C/A213R GBP-IANBD to normal and diabetic mouse lungs by inhalation to see if we could determine differences in luminal glucose concentration. This part of the study had a number of limitations. The distribution/fractionation of the sensor across the lung can be affected by respiratory rate which is reduced by anaesthesia and reportedly reduced by hyperglycaemia [32]. The emission wavelength of IANBD did not allow for imaging in whole tissue and it is close to that of lung tissue autofluorescence. Therefore, we used cryosections and spectral unmixing to generate images of the distal lung. The E149C/A213R GBP-IANBD fluorescence we detected in luminal regions of the lung indicated that, in accordance with other studies, nasal inhalation was sufficient to get the sensor to the distal lung [33–35]. However, we could not clearly demonstrate that fluorescence was associated with ASL. Whilst we were able to show higher sensor fluorescence in hyperglycaemic compared to normal mice we cannot be certain that the glucose was derived from ASL and not plasma/interstitial fluid leak from the process of cryo-sectioning. As E149C/A213R GBP-IANBD output from normal and hyperglycaemic sections were similar to that using sensor pre-bound with 10 mM glucose indicated that this may have been the case. It is also conceivable that glucose in the ASL of mice is higher than that reported for human airway secretions [36]. Nevertheless, our data provide proof of concept for the potential use of such glucose sensors in the lungs *in vivo*.

In conclusion, we have developed a longer wavelength glucose sensor E149C/A213R GBP-IANBD that can be used to directly image and measure glucose concentration in ASL *in vitro*. We have shown that the sensor can be delivered to the lung lumen and that the sensor reports differences in glucose concentration in the lungs of normoglycaemic vs hyperglycaemic mice although it remained unclear if the glucose was derived solely from ASL *in vivo*. Further detailed validation is now required but we propose that this sensor provides a novel tool for development to measure changes in glucose concentration in models of lung diseases.

## Supporting information

S1 Fig *E*. *coli* GBP with the E149 residue highlighted in red and the A213 residue highlighted in orange. a- The protein in its open conformation in the absence of glucose. b- The protein in its closed conformation in the absence of glucose. Figure b has been rotated 90° along the y-axis compared to figure a.
(TIF)
