# Supplementary figures and images for: A modified fluorescent sensor for reporting glucose concentration in the airway lumen
Source: PLoS One. 2021 Jul 9;16(7):e0254248. doi: 10.1371/journal.pone.0254248 (PMC8270177; doi:10.1371/journal.pone.0254248)

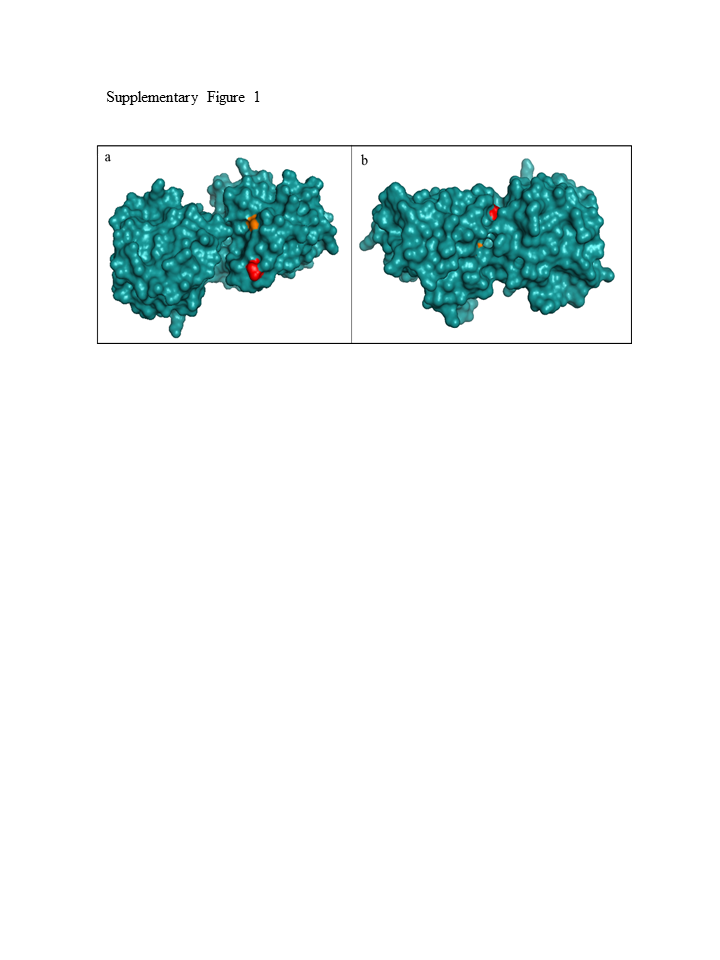

Supplement: S1 Fig — a- The protein in its open conformation in the absence of glucose. b- The protein in its closed conformation in the absence of glucose. Figure b has been rotated 90° along the y-axis compared to figure a. (TIF) [file pone.0254248.s001.TIF]
